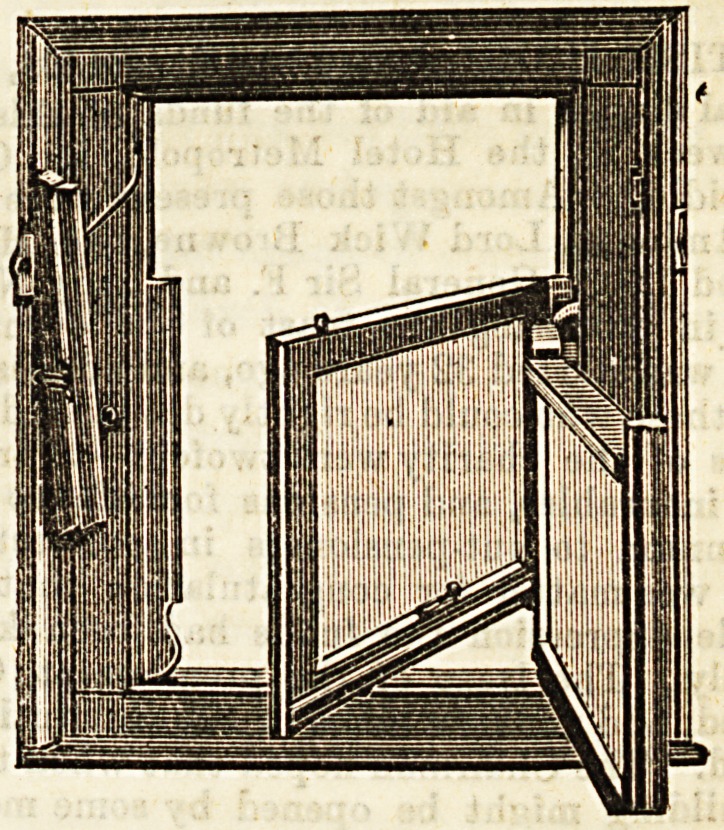# The "Braloo" Window

**Published:** 1893-06-03

**Authors:** 


### THE INSTITUTIONAL WORKSHOP.

### PRACTICAL DEPARTMENTS.

### THE "BRALOO" WINDOW.

A new idea in the matter of window frames has been lately patented by Messrs. Rindesland and Pattenden, Harrow Road, W., of which the objects are shown by the illustrations we give below. Its main intention is to facilitate cleaning operations, and thus to prevent accidents to life and limb, which have too often happened in the past. The sight of some unfortunate housemaid balancing herself half in and half out of a window four or five stories high used to be far too frequent; now, fortunately, public opinion has declared against the danger of such acrobatic performances. The inconveniences of windows which can only be cleaned from within by such means, or by the use of ladders from without, are obvious, but in spite of the opinion held by some people that sash windows, architecturally speaking, are "a mass of blunders," they yet remain the most popular, and so far as ventilation is concerned are undoubtedly the best. Any plan which will obviate the cleaning difficulty, and yet retain the sash form, will be a distinct boon, and in many ways the new invention of which we are speaking seems to fulfil these conditions.

As will be seen by the first drawing, the "Braloo" window has just the appearance of an ordinary sash window when closed, and for ordinary use is treated in the same way, with the additional advantage that it can be opened two or three inches top and bottom, one or both, and so fixed that it cannot be opened further from without. The sash lines are enclosed in the frames, these being made with doors so that the lines can be easily repaired.

The second illustration shows the window opened for cleaning. This can be easily done by anyone inside the room, without difficulty or danger. The sashes are brought into the position shown in the drawing without any trouble, and are quickly replaced.

The idea is certainly a good one. The only fault we have to find with the construction of these window frames is that they are rather clumsy in appearance. The woodwork is too heavy in proportion to the size of the window, this being apparently necessitated by the unavoidable number of hinges, &c., which are required to enable the sashes to be easily moved into the various positions. The invention is a very young one as yet, however, and Messrs. Rindesland and Pattenden will probably find means to remedy this matter in some degree, when the "Braloo" window will become of real practical value.

The illustrations are used by kind permission of Messrs. Rindesland and Pattenden.

## Figures and Tables

**Figure f1:**
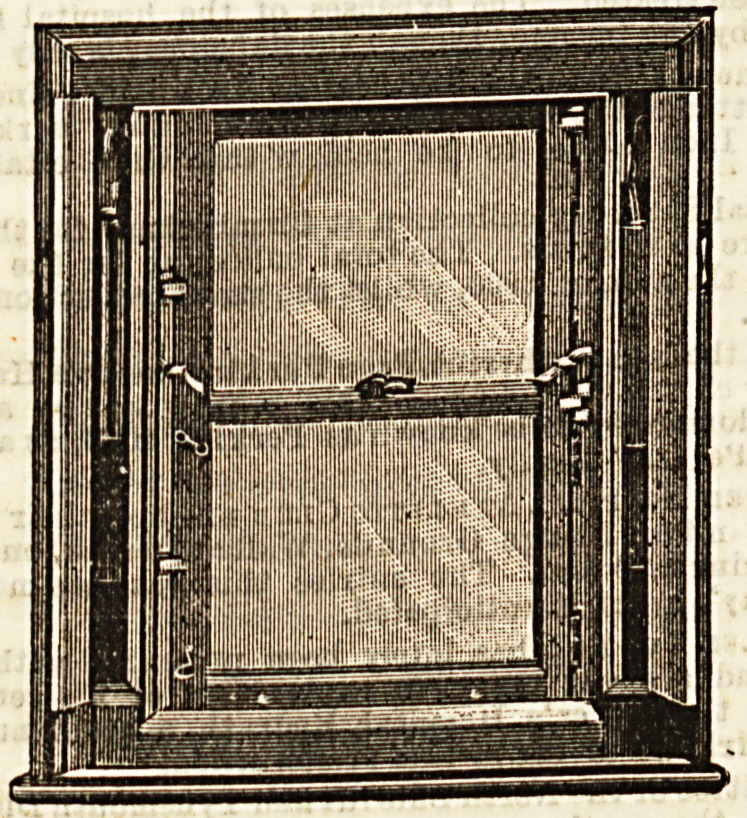


**Figure f2:**